# PHARMACOLOGICAL TREATMENTS FOR FATIGUE IN INFLAMMATORY BOWEL DISEASE PATIENTS: AN INTEGRATIVE REVIEW

**DOI:** 10.1590/S0004-2803.24612025-081

**Published:** 2026-01-09

**Authors:** Tayane MORAIS, Genalva COUTO, Raquel ROCHA, Genoile SANTANA

**Affiliations:** 1 Universidade do Estado da Bahia, Pós-graduação stricto sensu em Ciências Farmacêuticas, Salvador, Bahia, Brasil.; 2 Universidade Federal da Bahia, Escola de Nutrição, Salvador, Bahia, Brasil.

**Keywords:** Inflammatory bowel disease, crohn’s disease, ulcerative colitis, fatigue, drug therapy, chronic disease, Doença inflamatória intestinal, doença de Crohn, retocolite ulcerativa, fadiga, terapia medicamentosa, doença crônica

## Abstract

**Background:**

In patients with Inflammatory Bowel Disease (IBD), fatigue is a debilitating problem and may be associated with sleep disturbance, anxiety, depression, anemia, use of systemic steroids and active phase of the disease. In addition, fatigue also affects the working conditions of these patients, as it is associated with absenteeism and is a reason for time off work, surpassing medical appointments and abdominal pain. Currently, there are no well-established pharmacological therapies for fatigue, making it a subject of growing research interest.

**Objective:**

This study aimed to conduct an integrative review of pharmacological treatments for fatigue in patients with IBD.

**Methods:**

Inclusion criteria included full articles published from January 1, 2017 to December 31, 2024. Eligible studies had to include fatigue assessment as a primary objective and discuss pharmacological treatments for fatigue in IBD patients.The databases used were PubMed, Lilacs, SciElo and Cochrane and the descriptors were (inflammatory bowel disease) AND (fatigue) AND (drug therapy).

**Results:**

Total of 294 studies were identified, of which ten met the inclusion criteria, comprising 2,935 patients (1,664 with Crohn’s disease, 1,215 with ulcerative colitis, and 56 with irritable bowel syndrome). Vitamin B12 has not demonstrated efficacy in alleviating fatigue in IBD patients. High doses of oral thiamine reduce fatigue, but studies using a dose of 300 mg/day of thiamine have not shown the same effect. Fatigue symptoms have been reduced with the use of vedolizumab, upadacitinib and modafinil. Studies assessing pharmacological treatments for fatigue in IBD remain limited, and available data are still insufficient. Establishing effective pharmacological therapies for fatigue in these patients may lead to better physical and emotional well-being, enhanced social interactions and employability, and reduced financial burdens associated with fatigue management. Further randomized clinical trials and systematic reviews are necessary to advance the understanding of pharmacological interventions for fatigue in IBD.

## HIGHLIGHTS

•Even though fatigue is a prevalent, debilitating, and burdensome symptom in patients with inflammatory bowel disease (IBD), few studies have evaluated pharmacological treatments. This integrative literature review found that high doses of thiamine, modafinil, vedolizumab, and upadacitinib reduced fatigue symptoms, while vitamin B12 and 5-hydroxytryptophan supplementation had no significant effect. Additional randomized controlled trials and systematic reviews with meta-analysis are needed to establish effective pharmacological treatments for this condition.

## RESUMO

**Contexto:**

Em pacientes com Doença Inflamatória Intestinal (DII), a fadiga é um problema debilitante e pode estar associada a distúrbios do sono, ansiedade, depressão, anemia, uso de esteroides sistêmicos e fase ativa da doença. Além disso, a fadiga também afeta as condições de trabalho desses pacientes, pois está associada ao absenteísmo e é motivo de afastamento do trabalho, superando consultas médicas e dores abdominais. Atualmente, não existem terapias farmacológicas bem estabelecidas para a fadiga, tornando-a um assunto de crescente interesse em pesquisa.

**Objetivo:**

Este estudo teve como objetivo realizar uma revisão integrativa dos tratamentos farmacológicos para fadiga em pacientes com DII.

**Métodos:**

Os critérios de inclusão incluíram artigos completos publicados de 1º de janeiro de 2017 a 31 de dezembro de 2024. Os estudos elegíveis tiveram que incluir a avaliação da fadiga como objetivo principal e discutir tratamentos farmacológicos para fadiga em pacientes com DII. As bases de dados utilizadas foram PubMed, Lilacs, SciElo e Cochrane e os descritores foram (inflammatory bowel disease) AND (fatigue) AND (drug therapy).

**Resultados:**

Foram identificados 294 estudos, dos quais dez preencheram os critérios de inclusão, compreendendo 2.935 pacientes (1.664 com doença de Crohn, 1.215 com colite ulcerativa e 56 com síndrome do intestino irritável). A vitamina B12 não demonstrou eficácia no alívio da fadiga em pacientes com DII. Altas doses de tiamina oral reduzem a fadiga, mas estudos usando uma dose de 300 mg/dia de tiamina não mostraram o mesmo efeito. Os sintomas de fadiga foram reduzidos com o uso de vedolizumabe, upadacitinibe e modafinila. Estudos que avaliam tratamentos farmacológicos para fadiga na DII permanecem limitados e os dados disponíveis ainda são insuficientes. O estabelecimento de terapias farmacológicas eficazes para fadiga nesses pacientes pode levar a um melhor bem-estar físico e emocional, a interações sociais e à empregabilidade aprimoradas, além de reduzir os encargos financeiros associados ao manejo da fadiga. Mais ensaios clínicos randomizados e revisões sistemáticas são necessários para avançar na compreensão das intervenções farmacológicas para fadiga na DII.

## INTRODUCTION

Inflammatory bowel disease (IBD) is a chronic inflammatory condition that results from the interaction between genetic and environmental factors that influence immune responses and one’s quality of life. Recent studies also indicate the involvement of short-chain fatty acids (main metabolites produced through anaerobic fermentation of indigestible polysaccharides by the intestinal microbiota) in the development of inflammatory bowel diseases^1^ ^,^ ^2^. IBD affects seven million individuals worldwide and manifests through symptoms such as abdominal pain, hematochezia, tenesmus, diarrhea, and rectal bleeding, often leading to irreversible damage and intestinal dysfunction^4^ ^,^ ^5^. The most common forms of IBD are ulcerative colitis (UC) and Crohn’s disease (CD)^3^.

The overall prevalence of fatigue in Crohn’s disease is 47% (95% confidence interval, 41-54%). The prevalence of fatigue varies significantly according to the disease state, being present in 72% of active cases and 47% in disease remission. Fatigue is twice as common in patients with IBD than in healthy controls, and occurs in up to 50% of patients with IBD at the time of diagnosis, being more common in CD than in UC^6^. The etiology of fatigue in IBD is multifactorial and may be associated with inflammation, anemia, nutrient deficiencies, medications, and microbiota^7^ ^,^ ^8^. Fatigue is debilitating and costly for the patient and the health system.

Fatigue in patients with IBD is associated with decreased physical functioning, mood disturbances, and low productivity, presenting symptoms of physical and mental exhaustion. Despite its widespread prevalence and significant debilitating consequences, fatigue in patients with IBD is often underestimated in clinical practice, and its treatment and management remain suboptimal^9^. As a consequence, pharmacological treatments for fatigue in patients with IBD have long been underexplored.

In patients with IBD, there are no well-established pharmacological therapies for fatigue, despite the growing number of studies exploring interventions that may improve patients’ living conditions^10^ ^,^ ^11^ ^,^ ^12^. Most of the interventions studied are behavioral or psychological, and only a few studies have evaluated the effects of medications^12^ ^-^ ^13^, for example, studies investigating whether correction of vitamin B1 deficiency anemia reduces physical fatigue and whether treatment with a central nervous system stimulant that blocks dopamine reuptake transport reduces mental fatigue in patients with IBD^14^. Given the lack of consensus on the treatment of fatigue in patients with IBD, the aim was to perform an integrative review of pharmacological treatments for fatigue in patients with IBD.

## METHODS

The methodological procedures were divided into six stages, addressing the following: identifying the topic and selecting the research question for the integrative review. Inclusion and exclusion criteria were established. The information to be extracted from the selected articles and categorizing the studies were determined. The research included in the integrative review was evaluated, the results were interpreted, and the review/knowledge synthesis was presented.

### First stage

In this stage, the topic was identified and the research question was selected for the integrative review.

The guiding question for this study was: what is the current scientific evidence, over the last seven years, on the pharmacological treatment of fatigue in patients with IBD?

### Second stage

In this stage, criteria for inclusion and exclusion of studies/sampling or literature search were established. The search followed the inclusion criteria: a) articles; b) available abstract; c) full text available in electronic format; d) article available in an open database. d) the period of the articles was from January 1, 2017, to December 31, 2024; e) articles available in English, Portuguese, and Spanish. Furthermore, if an article was identified in languages other than the three mentioned, the text would need to be translated by a professional qualified to translate scientific texts into the corresponding language. f) articles whose population consisted of people diagnosed with inflammatory bowel disease; g) the study design included randomized clinical trials, cohort studies, and prospective case series; h) the articles should have fatigue assessment among their main objectives and discuss pharmacological treatment for fatigue in patients with IBD; i) the articles needed to identify the scales used to assess fatigue in the IBD population. The exclusion criteria were texts unrelated to the topic. Conference abstracts, book chapters, letters to the editor, results and award reports, encyclopedias, and medical guidelines were also excluded.

### Third stage

This was the definition of the information to be extracted from the selected studies/study categorization. Following the data collection instrument validated by Ursi (2005)^15^, an electronic spreadsheet was used to organize research related to the topic, extracting information from the selected articles. The information collected included the year of publication, article title, authors, study type, country, language, journal, sample, and clarity in identifying the methodological design and study limitations.

### Fourth stage

The studies included in the integrative review were evaluated.

The selected articles were analyzed by reading their titles and abstracts. All articles that did not answer the research question or did not meet the inclusion criteria were excluded. The next stage involved a complete reading of the remaining articles after the previous stage.

The databases used for the search were PubMed, Cochrane, Scientific Electronic Library Online, and the Virtual Health Library. The descriptors were selected from the MeSH (Medical Subject Headings) vocabulary list published by the National Library of Medicine (United States of America). The literature search was conducted by a library specializing in systematic reviews, integrative reviews, and evidence-based practice. The descriptors used were “inflammatory bowel disease,” “fatigue,” and “drug therapy.” To refine and direct the database queries, the Boolean operator “AND” was used, thus filtering the most relevant information. Example of a search string: https://pubmed.ncbi.nlm.nih.gov/, and the terms “inflammatory bowel disease,” “fatigue,” and “drug therapy” were entered in the advanced field. 171 results were found (search date: January 31, 2024).

### Fifth stage

Results were interpreted.

### Sixth stage

Presentation of the review/knowledge synthesis.

## RESULTS

**FIGURE 1:**
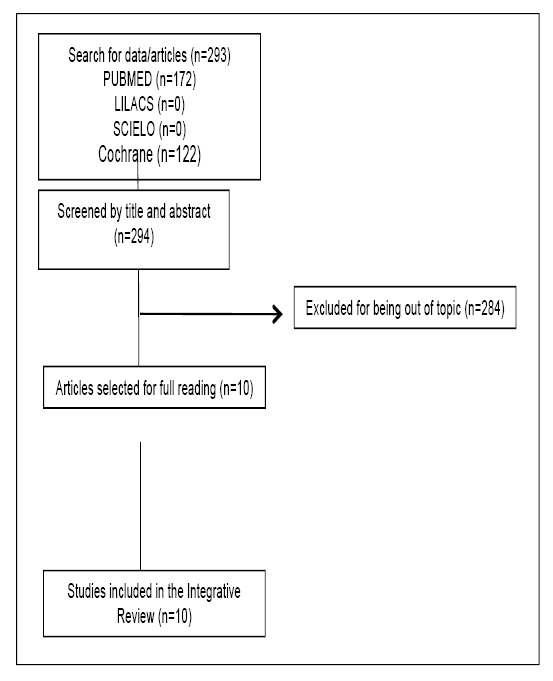
Selection process of articles that make up the integrative review (based on the PRISMA model).

The initial search including the descriptors identified 172 studies in PubMed and e 122 studies in COCHRANE. On Scientific Electronic Library Online (SCIELO) and Virtual Health Library (LILACS), no studies were found to be evaluated. According to the exclusion criteria, 284 articles outside the proposed theme, were identified after reading the title and abstract. Thus, the final sample of this review consisted of ten articles (Figure 1) totaling 2935 patients (1664 with CD and 1215 with UC and 56 patients diagnosed with irritable bowel syndrome). The collected data were organized in Tables 1 and 2.

**TABLE 1:** Articles selected to compose this integrative review.

Year	Authors	Article Title	Country	Language	Kind of study	Study period
2017	Scholten et al.	Overuse of vitamin B_12_ does not reduce fatigue in patients with IBS^a^ or IBD^b^: a randomized double-blind placebo-controlled trial	Netherlands	English	Randomized, double-blinding, placebo-controlled trial.	8 weeks
2020	Bager et al.	Randomized clinical trial: high-dose oral thiamine versus placebo for chronic fatigue in patients with quiescent IBD.	Denmark	English	Randomized, placebo-controlled clinical trial, crossover intervention design, triple blinding (participant, caregiver, and investigator)	12 weeks
2021	Moradi et al*.*	The effects of spirulina supplementation (*Arthrospira platensis*) on anthropometric indices, blood pressure, sleep quality, mental health, fatigue status, and quality of life in UC patients: a randomized, double-blind, placebo-controlled trial	Iran and UK	English	Randomized, double-blind, placebo- controlled study	8 weeks
2022	Bager et al.	Long-term maintenance treatment with 300 mg thiamine for fatigue in IBD patients: results from an open-label extension of the TARIF trial	Denmark	English	Randomised, open-label, placebo-controlled study. Long-term extension study of an early high-dose thiamine study.	39 weeks
2022	Truyens et al.	Effect of 5-hydroxytryptophan on fatigue in quiescent IBD: a randomized controlled trial	Belgium	English	Multicenter randomized, placebo-controlled study.	8 weeks
2023	Bager et al*.*	B vitamins, related vitamins, and metabolites in patients with quiescent inflammatory bowel disease and chronic fatigue treated with high-dose oral thiamine	Denmark	English	Randomized, double-blind, placebo-controlled crossover study	12 weeks
2023	Danese et al.	Induction and maintenance therapy with upadacitinib improves abdominal pain, bowel urgency, and fatigue in patients with ulcerative colitis: a post hoc analysis of phase 3 data	United States of America, Argentina, Australia, Austria, Belgium, Bosnia and Herzegovina, Brazil, Bulgaria, Canada, Chile, China, Colombia, Croatia, Czech Republic, Denmark, Egypt, Estonia, France, Germany, Greece, Hong Kong, Hungary, Ireland, Israel, Italy, Japan, Republic of Korea, Latvia, Lithuania, Malaysia, Mexico, Netherlands, Poland, Portugal, Puerto Rico, Romania, Russian Federation, Serbia, Singapore, Slovakia, Slovenia, South Africa, Spain, Sweden, Switzerland, Taiwan, Peru, Ukraine and United	English	A multicenter, randomized, double-blind, placebo-controlled induction study to evaluate the efficacy and safety of upadacitinib (ABT-494) in subjects with moderately to severely active ulcerative colitis.	52 weeks
2023	Steenholdt et al.	Trajectories of health-related quality of life and fatigue during vedolizumab therapy in inflammatory bowel disease	Denmark	English	Cohort study of patients with biologically refractory IBD treated with vedolizumab	54 weeks
2024	Moulton et al.	Modafinil for severe fatigue in inflammatory bowel disease: a prospective case series	United Kingdom	English	Prospective case series	24 months
2024	Ghosh et al.	Impact of induction and maintenance therapy with upadacitinib on health-related quality of life, fatigue, and work productivity in patients with moderately to severely active Crohn’s disease	United States of America, Argentina, Australia, Austria, Belgium, Bosnia and Herzegovina, Brazil, Bulgaria, Canada, Chile, China, Colombia, Croatia, Czech Republic, Denmark, Egypt, Estonia, France, Germany, Greece, Hong Kong, Hungary, Ireland, Israel, Italy, Japan, Republic of Korea, Latvia, Lithuania, Malaysia, Mexico, Netherlands, Poland, Portugal, Puerto Rico, Romania, Russian Federation, Serbia, Singapore, Slovakia, Slovenia, South Africa, Spain, Sweden, Switzerland, Taiwan, Peru, Ukraine and United Kingdom.	English	A Multicenter, Randomized, Double-Blind, Placebo-Controlled Maintenance and Long-Term Extension Study of the Efficacy	52 weeks

**IBS:** irritable bowel syndrome^a^. **IBD:** inflammatory bowel disease^b^.

**TABLE 2:** Evaluation of selected articles.

Year	Authors	Sample	Disease activity/scale	Limitations
2017	Scholten et al.	95 participants (56 with IBS^a^, 20 with CD^b^ and 19 with UC^c^), 70 (73.7%) were female and the mean age was 41 years.	Disease activity has not been evaluated.	A limitation of this study may be the cutoff point used for vitamin B_12_ deficiency. The present study used <150 pmol/L as the cutoff point for vitamin B12 deficiency, but there is no consensus on the cutoff point for vitamin B_12_ deficiency. Finally, serum vitamin B_12_ is not the most sensitive marker for measuring vitamin B_12_ status, which could indicate vitamin B_12_ deficient subjects in the study.
2020	Bager et al.	40 patients (20 with CD and 20 with UC). Group 01 had 20 patients (mean age 38 years, and 17 (85%) were female). Group 02 had 20 patients (mean age 36 years and 18 (90%) were female).	All patients were in disease remission.(HBICD^d^ e CSCAI^e^)	Young patient cohort, single-center design, and exclusion criteria (patients with actual chronic disease were excluded without defining an epidermal growth factor receptor cutoff, but patients with rheumatoid arthritis were included).
2021	Moradi et al.	73 patients with UC. The group that used Spirulina had 36 patients (mean age 37.8 years and 18 (50%) were female). The placebo group had 37 patients (mean age 39.5 years and 20 (54.0%) were female	Light to moderate active(SSCCAI^f^)	The study did not evaluate the dose-dependent efficacy of spirulina supplementation, which means that one cannot infer sucha relationship. Intervention time was potentially insufficient to cause anthropometric changes.
2022	Bager et al*.*	40 patients, 35 (87.5%) were female and the mean age was 37 years (20 with CD and 20 with UC).	All patients were in disease remission.(HBICD and CSCAI)	Young patient cohort, single-center design, and exclusion criteria (patients with actual chronic disease were excluded without defining an epidermal growth factor receptor cutoff, but patients with rheumatoid arthritis were included).
2022	Truyen et al*.*	166 patients, 94 (56.6%) were female, and the mean age was 39 years. 84 patients had CD, and 82 had a diagnosis of UC	All patients were in disease remission. (SHBI^g^)	Remission was defined based on clinical criteria and biochemical indices; however, ongoing subclinical inflammation, which could be related to fatigue, has not been evaluated.Sleeping quality was unrated, so it is impossible to relate fatigue burden to sleep quality in this study cohort.
2023	Bager et al*.*	40 patients (20 with CD and 20 with UC). Group 01 had 20 patients (mean age 38 years, and 17 (85%) were female). Group 02 had 20 patients (mean age 36 years and 18 (90%) were female).	All patients were in disease remission.(HBICD^d^ e CSCAI^e^)	The study itself, regarding the post-analysis of B vitamins and related vitamins, has some limitations. The study included primarily women. The sample size seemed appropriate, as the primary results were clear. Furthermore, the control group was very similar to the intervention group. However, a larger sample size may have revealed more significant results in this B vitamin substudy.
2023	Danese et al.	Total de 988 pacientes com diagnóstico de colite ulcerativa, of which 372 (37.65%) were female. The average age of the patients was 41.5 years.	Moderately to severely active ulcerative colitis, defined by an adapted Mayo score of 5 to 9 points and endoscopic subscore of 2 to 3 (confirmed by central reader)	Limitations of this study include the generalizability of the findings to patients with mild colitis and the lack of granularity in the measurement of abdominal pain and bowel urgency. Therefore, it is difficult to assess patients who may benefit most from a reduction in pain or urgency.
2023	Steenholdt et al.	The study population comprised 79 patients with IBD (40% women), but fatigue was only assessed in one subgroup (30 patients, 21 with ulcerative colitis and 9 with Crohn’s disease).	Clinical remission in patients with Crohn’s disease was defined as Harvey-Bradshaw Index (HBI) < 5, and in ulcerative colitis as Simple Clinical Colitis Activity Index (SCCAI) ≤ 2.	The main limitation of the study is the limited number of patients and the retrospective collection of recorded data. Furthermore, fatigue assessments were implemented later and this subcohort was therefore small and the findings exploratory. Disease activity was assessed objectively after 1 year of VDZ therapy, or at treatment failure, preferably by endoscopy, but with a minority being assessed by other measures reflecting clinical practice.
2024	Moulton et al	Total of 10 patients - 8 females and 2 males in the series, mean age was 33.7 years (SD, 8.4). 8 patients were diagnosed with Crohn’s disease and 2 with ulcerative colitis.	Eight patients had Crohn’s disease and 2 patients had ulcerative colitis. Does not mention a scale to assess disease activity.	Small sample size, limited duration of follow-up, lack of randomization, lack of measurement of fatigue impact, lack of follow-up fecal calprotectin measurement, lack of control group and lack of blinding, and require replication in a randomized controlled trial.
2024	Ghosh et al.	A total of 1,523 patients (all diagnosed with Crohn’s disease) were evaluated for induction and maintenance of Crohn’s disease. Of these, 845 were male. The age ranged from 18 to 75 years.	Patients with active disease were used as eligibility criteria. The study observed disease remission with the drug upadacitinib. Remission was assessed by endoscopy (Simplified Endoscopic Score for Crohn’s Disease - SES-CD) and clinical remission was defined based on average daily stool frequency and average daily abdominal pain score (CDAI). Clinical remission by CDAI was defined as CDAI <150.	The trials had certain limitations as the findings cannot be generalized to real-world settings and to patients with milder CD who were not represented in the sample.
TABLE 2. Evaluation of selected articles (continued).				
Year	Authors		Scales to measure fatigue	Main results related to fatigue	
2017	Scholten et al.		Checklist Individual Strength.Visual Analogue Scale.Fatigue Impact Scale.	Based on the study, it was not possible to confirm the expected unconventional effect onfatigue after 8 weeks of excess treatment with oral vitamin B_12_ (there was no significant reduction in scores based on the questionnaires used to measure fatigue, when compared to placebo).	
2020	Bager et al.		Inflammatory Bowel Disease Fatigue Scale (IBD-F)	The study showed that high doses of oral thiamine had a significant beneficial effect in reducing fatigue after 4 weeks. Cross-analysis identified an average reduction of 4.5 points in scores on the questionnaire used to assess fatigue after thiamine.	
2021	Moradi et al.		Fatigue Severity Scale (FFS)	Despite the study identifying an improvement in quality of life after the intervention period with the use of spirulina supplementation but there was no significant reduction in fatigue.	
2022	Bager et al.		Inflammatory Bowel Disease Fatigue Scale (IBD-F)	No beneficial effect was identified in reducing fatigue after using thiamine 300 mg for 12 weeks and following these patients for 6 months, as 66% reached a normal level of fatigue in relation to the period before the intervention.	
2022	Truyens et al.		Chronic Illness Fatigue Therapy (FACIT-F)	5-Hydroxytryptophan did not reduce fatigue in patients with IBD when compared with placebo after 8 weeks of intervention.	
2023	Bager et al.		Inflammatory Bowel Disease Fatigue Scale (IBD-F)	Flavin mononucleotide (FMN) concentration was lower in patients with chronic fatigue compared with patients without fatigue (p = 0.02). Patients with chronic fatigue who reported a positive effect on fatigue after 4 weeks of high-dose thiamine treatment had a statistically significantly lower level of riboflavin after thiamine treatment (p = 0.01).	
2023	Danese et al.		Chronic Illness Fatigue Therapy (FACIT-F)	Significant and clinically meaningful improvements in fatigue were observed in patients treated with upadacitinib at week 2, fatigue improved further by week 8 of induction, and improvements were sustained through 52 weeks of maintenance treatment compared with placebo. The results of this study showed that upadacitinib has a long-term positive impact on the relief of abdominal pain, bowel urgency, and fatigue among patients with moderately to severely active ulcerative colitis when compared with placebo.	
2023	Steenholdt et al.		Visual Analogue Scale - Fatigue	Quality improves rapidly and predicts subsequent meaningful clinical-objective efficacies of vedolizumab at the end of induction and one year. Fatigue improves slowly after remission is achieved.	
2024	Moulton et al		IBD Fatigue Assessment Scale (IBD-FAS)	Six of 10 patients had a 50% or greater improvement from baseline IBD-FAS score. Although all 10 patients were severely fatigued at baseline, only 2 patients were still in the severe fatigue range after treatment. Modafinil dramatically improved IBD fatigue, suggesting that centrally available dopamine deficiency may be a significant cause of IBD fatigue.	
2024	Ghosh et al.		Chronic Illness Fatigue Therapy (FACIT-F)	In patients with active Crohn’s disease, upadacitinib treatment relative to placebo significantly improved fatigue, quality of life, and work productivity as early as Week 4. These effects were sustained through 52 weeks of maintenance.	

Irritable bowel syndrome^a^. CD: crohn’s disease^b^. UC: ulcerative colitis^c^. HBICD: harvey-bradshaw index for crohn’s disease^d^. CSCAI: clinical simple colitis activity index^e^. SSCCAI: scale-simple clinical colitis activity index^f^. SHBI: scale-harvey-bradshaw index^g^.

Regarding the diagnosis of inflammatory bowel disease, in the article by Scholten et al. (2017)^16^, the diagnosis was by histopathologic confirmation. In the article by Moradi et al. (2021)^17^, the diagnosis was via colonoscopy, clinical records, and pathology. Danese et al. (2023)^18^ and Ghosh et al. (2024)^19^, data for the diagnosis of Crohn’s disease and ulcerative colitis were obtained from medical records and to assess remission, the Simple Clinical Colitis Activity Index (for ulcerative colitis) and Harvey Bradshaw Index (for Crohn’s disease) were used for CD. At screening, patients had to have confirmed biochemical remission, defined by a C-reactive protein level of <10 mg/L and a fecal calprotectin level of <250 mg/kg. Steenholdt et al. (2023)^20^ obtained data on the diagnosis of IBD via electronic medical records. Moulton et al. (2024)^17^ do not report how the IBD diagnosis was made but state that participants were IBD patients being monitored at St Mark’s Hospital (London, United Kingdom)^17^ Bager et al. (2020)^12^, Bager et al. (2022)^22^, Bager et al. (2023)^23^ and Truyens et al. (2020)^24^ do not provide information on how the IBD diagnosis was made.

The subjective and multifaceted nature of fatigue makes it difficult to understand and measure. Quantifying fatigue is challenging due to the lack of a consensus framework and terminology. Currently, self-report measures are used for the assessment of fatigue. Instruments can be categorized based on the number of dimensions of the disease measured: unidimensional instruments record a single fatigue component and multidimensional instruments divide fatigue into, for example, physical and mental or cognitive dimensions^8^ ^,^ ^25^ ^,^ ^26^. Of the ten articles selected, six scales were identified to measure fatigue. Two of the articles studied assessed fatigue using the visual analogue fatigue scale. This is a unidimensional scale, which measures the intensity of fatigue. Higher scores represent greater severity or intensity of fatigue^27^.

Four articles used the Inflammatory Bowel Disease Fatigue Scale (IBD-F). The IBD-Fatigue scale was developed, tested, and validated for patients with inflammatory bowel disease, and is the first specifically for this population. This scale consists of items generated specifically based on issues important to people with fatigue in IBD and was found to be valid and reasonably reliable in initial testing^25^. Three articles used the Functional Assessment of Chronic Illness Therapy - Fatigue (FACIT-F). This scale is a 40-item instrument that assesses self-reported fatigue and its impact on daily activities and functions. This scale has already been validated to measure fatigue in an inflammatory bowel disease population^28^. One of the articles measured fatigue using the Fatigue Severity Scale (FFS). This instrument measures the perception of the level of fatigue in different everyday situations (physical functioning, exercise, work, family or social life), while also allowing the assessment of fatigue in various diseases (e.g., depression, multiple sclerosis, systemic lupus erythematosus, chronic fatigue syndrome and inflammatory bowel disease)^29^.

Some heterogeneity among the ten reviewed articles should be considered. The study designs varied, including three randomized double-blind placebo-controlled trials (single-center), one randomized triple-blind trial, one randomized open-label placebo-controlled study, three randomized multicenter placebo-controlled studies, one cohort study, and one prospective case study. Regarding fatigue severity, differences among the articles were observed, as follows: The study by Scholten et al. (2017)^16^ selected only patients with severe fatigue. Bager et al. (2020, 2022 and 2023)^12^ ^,2,^ ^24^ selected patients with moderate and severe fatigue. Moradi et al. (2021)^17^ excluded patients with severe ulcerative colitis, leaving only mild and moderate. The article selected patients with fatigue for the study (calculated the fatigue score between the control group and the group that used the treatment, but did not classify fatigue as mild, moderate, or severe). Danese et al. (2023)^18^ selected only patients with moderately to severely active ulcerative colitis for the study. They did not classify fatigue as mild, moderate, or severe. Steenholdt et al. (2023)^20^ investigated the evolution of health-related quality of life and fatigue throughout treatment with vedolizumab and according to clinical remission at the end of induction and clinical and objective remission at 1 year. Patients with IBD treated with due to luminal disease were included in the study (patients with fistulizing Crohn’s disease were excluded). Although the study had 79 participants, fatigue was only assessed in a subgroup with 30 participants. At the beginning of treatment with vedolizumab, the Visual Analog Scale-F fatigue was a mean of 4.6, SD 2.8 (which is considered moderate fatigue). Moulton et al. (2024)^21^ included only patients with severe fatigue (mean Inflammatory Bowel Disease Fatigue Scale score was 16.0 (SD, 1.7) out of 20). Ghosh et al. (2024)^19^ selected only patients diagnosed with moderately to severely active Crohn’s disease. They did not classify fatigue intensity.

To date, few researchers have evaluated the effectiveness of different interventions to manage IBD fatigue. Most research is directed towards investigating risk factors for fatigue in patients with IBD and symptom management. There are few studies investigating drug therapies for fatigue symptoms in patients with IBD (Table 1). In the reports of the ten selected articles, promising results are not presented (Table 2).

### Spirulina

The biomass of the cyanobacteria Arthrospira platensis is a promising food source of biologically active substances with pharmacological activity^30^. It has been used for attention-deficit hyperactivity disorder (ADHD), allergic rhinitis, hypertension, diabetes, stress, fatigue, anxiety, and depression^30^. The biomass of the cyanobacteria Arthrospira platensis is a promising food source of biologically active substances with pharmacological activity^31^.

The study by Johnson and colleagues (2015) tested the hypothesis that spirulina may increase people’s resistance to mental and physical fatigue^32^. It was a randomized, double-blind, placebo-controlled study in men. After one week, a dose of 3 g/day of spirulina resulted in a small but statistically significant increase in exercise performance (calories burned in 30 minutes of exercise on a cross trainer). A math-based mental fatigue test showed improved performance four hours after the first supplementation and also eight weeks later^32^.

Gurney and Spendiff (2020)^33^ investigated the responses of spirulina supplementation on hemoglobin and oxygen uptake during seated arm cycling exercise (a randomized, double-blind crossover study in which eleven men not trained in arm cycling ingested 6 g/day of spirulina or placebo for seven days). The results of the Gurney and Spendiff (2020)^33^ study are consistent with previous literature, according to which spirulina supplementation caused significant increases in hemoglobin. Spirulina supplementation significantly reduced oxygen uptake and heart rate during submaximal arm cycling exercise, allowing for an increase in oxygen uptake during an incremental fatigue test^33^.

In 2021, a randomized, double-blind, placebo-controlled study was carried out to evaluate the effects of spirulina (Arthrospira platensis) with the primary objectives of evaluating anthropometric data, blood pressure, sleep quality, mental health, fatigue status and quality of life in patients with UC. The study’s exclusion criteria included patients with heart, liver, kidney, and oncological diseases, conditions that could explain fatigue in these patients^7^.

Eighty participants with UC were randomly allocated to receive 1 g/day (two 500 mg capsules) of spirulina (n=40) or placebo (n=40). All patients were instructed to continue their usual physical activity, dietary intake, and their current medication regimen during the follow-up period. No significant reduction was observed in anxiety, depression, or fatigue scores; however, there was an improvement in sleep disorders and stress levels^17^.

### 5-hydroxytryptophan supplementation

5-Hydroxytryptophan (5-HTP) is the precursor compound for serotonin biosynthesis. The oral absorption of 5-HTP is close to 100%, and, unlike serotonin, it freely crosses the blood-brain barrier. 5-HTP has been used as a dietary supplement for many years in cases of anxiety and depression. Recent studies have shown that 5-HTP suppresses pro-inflammatory mediators and is effective in some inflammatory diseases, such as arthritis and allergic asthma^34^.

Yamamoto and Newsholme (2024)^35^ studied the reduction of central fatigue through the inhibition of the L-system transporter for tryptophan uptake. They used genetically analbuminemic (NAR) rats of the Nagase breed, which were subjected to fatigue. Administration of branched-chain amino acids (BCAA) before exhaustive exercise resulted in a post-fatigue decrease in tryptophan uptake (-22%, *P*<0.05) and 5-hydroxytryptophan (5-HTP) uptake (-29%, *P*<0.01) in isolated striatal synaptosomes when compared to saline administration. At the same time, NARs receiving BCAA or 2-aminobicyclo [2,2,1] heptane-2-carboxylic acid (BCH, a specific inhibitor for the L-system transporter) had a significantly prolonged running time to exhaustion (by two-fold) compared to those receiving saline or albumin treatments. When classified by running time, an interesting finding was that combining data for BCAA and BCH treatments in the longest-running NAR group (Group B) led to a significant decrease in synaptosomal tryptophan and 5-HTP, similar to the reduction observed with BCAA alone. These levels were lower than those observed in NARs in the shortest-running group (Group A) across all treatments. These results support the view that an activated serotonergic function may be involved in central fatigue, which can be diminished by inhibition of the L-system transporter^36^.

Because 5-HTP crosses the blood-brain barrier, it has been explored as a treatment for depression and fatigue in various disease states^36^. In 2022, the effect of 5-HTP supplementation on fatigue in IBD patients in remission was investigated^17^. Research was conducted based on the hypothetical link between low serum tryptophan concentrations and fatigue. Thus, a placebo crossover study was performed to study the effect of oral ingestion of an essential amino 5-HTP, the precursor of serotonin, on fatigue scores in patients with IBD in deep clinical and biological remission^24^. Fatigue was assessed using Visual Analog Scale and Fatigue Impact Scale for Chronic Diseases Individuals with comorbidities such as obstructive pulmonary pathology, neoplasia, anemia, iron deficiency, vitamin B12 deficiency, and the use of corticosteroids in the past eight weeks (factors that could explain the fatigue in these patients) were excluded from the study^24^.

Patients were cross-treated with 100 mg of oral 5-HTP or placebo twice daily for two consecutive 8-week periods. Despite a significant increase in serum 5-HTP and serotonin levels, oral 5-HTP did not modulate IBD-related fatigue better than placebo. This study also evaluated whether 5-HTP reduced anxiety, depression, and stress. No differences were observed between the placebo group and the 5-HTP group^24^.

### Vitamin supplementation

Vitamin B12 is often used to improve cognitive function, depressive symptoms and fatigue. Considering the association of subclinical serum levels of vitamin B12 with declines in cognitive function, depressive symptoms and fatigue, vitamin B12 supplementation appears to be a promising treatment option and is generally prescribed by physicians in clinical practice for fatigue situations^37^.

Vitamin B12 deficiency is a common cause of fatigue in IBD and is treated with oral or parenteral vitamin B12. In 2017, an 8-week, double-blind, placebo-controlled clinical trial was conducted to evaluate the effect of high-dose (1,000 mg/d) oral vitamin B12 supplementation on reducing fatigue in patients with IBD and irritable bowel syndrome. Only patients with severe fatigue were selected for the study, but there were no exclusion criteria or identification of comorbidities that could be associated with fatigue^16^. There was an assessment of blood levels of vitamin B12 before and after the intervention period. The study does not provide information on the number of patients with vitamin B12 deficiency among those diagnosed with IBD. There was a significant increase in vitamin B12 levels of 254 pmol/l (±138 pmol) in the intervention group compared to an increase of 13 pmol/l (±72 pmol) in the placebo group. The study showed that fatigue did not improve after eight weeks of treatment. Patients were advised to maintain their regular diet and physical activity levels as before the study, as well as the same physical activities that occurred before the research). The authors concluded that there is no clinical indication for treatment with excess oral vitamin B12 in this group of patients^16^.

In 2013, Italian researchers investigated the effects of thiamine on fatigue in IBD patients. It was an open pilot study with eight patients with UC and four patients with CD. All patients were assigned to receive high doses of oral thiamine. The dosage varied according to body weight, ranging from 600 mg/day (for 60 kg) to 1,500 mg/day (for 90 kg). Ten out of twelve patients showed complete regression of fatigue, while the remaining two experienced near-complete regression. The chronic fatigue syndrome scale was used to assess fatigue before using thiamine and after 20 days of use. Blood thiamine levels were also measured before the treatment and 20 days after starting therapy. This study suggested that fatigue in IBD manifests as a thiamine deficiency, probably due to a dysfunction of active thiamine transport within cells or structural enzymatic changes, which would be resolved with high doses of thiamine^38^.

Based on the pilot study mentioned above, a randomized, double-blind, placebo-controlled crossover study was performed to evaluate high doses of thiamine in the treatment of fatigue in patients with IBD. The patients included in the research had quiescent IBD, severe fatigue, and no other explanation for their fatigue. Patients were allocated 1:1 to 1. high-dose oral thiamine for four weeks, followed by four weeks of washout and then four weeks of oral placebo, or 2. oral placebo for four weeks, followed by four weeks of washout and then four weeks of high-dose oral thiamine. Fatigue was measured using the inflammatory bowel disease fatigue questionnaire. The primary outcome was improvement (≥3 points) in fatigue after four weeks of thiamine use. Forty patients were enrolled between November 2018 and October 2019. Cross-over analysis showed a mean reduction of 4.5 points (95%CI 2.6-6.2) in fatigue after thiamine compared to a mean increase of 0.75 points (95%CI -1.3-2.8; *P*=0.0003) after placebo. Additionally, 55% of group 1 and 75% of group 2 showed an improvement of ≥3 points while taking thiamine, compared to 25% of group 1 and 35% of group 2 while on placebo. Only mild side effects were detected, and the treatment was well tolerated. There was a significant beneficial effect of high doses of oral thiamine on fatigue symptoms in IBD patients^12^.

Researchers have continued to study the use of thiamine to treat fatigue in IBD. They conducted a randomized, open-label, controlled trial as a long-term extension study of previous research. Patients were randomized 1:1 to receive oral thiamine 300 mg or no thiamine for 12 weeks. Subsequently, patients were allowed to self-medicate with over-the-counter oral thiamine for six months. The researchers found no beneficial effect in reducing fatigue after using thiamine at a daily dose of 300 mg for 12 weeks, following treatment with high doses of thiamine^22^. According to these two studies on thiamine supplementation to treat fatigue in patients with IBD, a reduction in fatigue symptoms was only observed with the use of high doses of thiamine.

Bager et al. (2023)^23^ analyzed plasma samples obtained in a randomized clinical trial (Bager et al. 2021^12^) and aimed to compare the levels of vitamins B1, B2, B3, and B6, and their related vitamins and metabolites in patients with IBD, with or without chronic fatigue and with or without the effect of high-dose oral thiamine for chronic fatigue. Blood samples from patients with fatigue were collected before and after thiamine exposure and only once for patients without fatigue. Flavin mononucleotide and riboflavin concentrations were associated with chronic fatigue in patients with quiescent IBD. The levels of other B vitamins and metabolites were not significantly different between the investigated groups or related to the effect of thiamine intervention.

### Modafinil

Modafinil, a central nervous system stimulant also known as diphenylmethylsulfinyl acetamide, has been shown to be an effective agent against daytime fatigue caused by narcolepsy and obstructive sleep apnea. The mechanism of action of modafinil is not yet fully understood; however, it is known to interact with the dopamine and norepinephrine systems, likely resulting in the inhibition of the reuptake of the respective catecholamines. As a result of the increase in general cortical activity caused by this drug, a great deal of research has been done to understand its potential for use as a cognitive enhancer and neuroprotective agent, as well as its properties as a stimulant and fatigue reducer^39^.

Moulton et al. (2024)^21^ studied the use of modafinil for severe fatigue in patients with inflammatory bowel disease. It was a prospective case series of ten patients. At baseline, the mean Inflammatory Bowel Disease Fatigue Scale score was 16.0 (SD, 1.7) out of 20. After modafinil treatment, the mean Inflammatory Bowel Disease Fatigue Scale score was 6.7 (SD=3.0), representing a mean improvement of 58.1% from baseline (paired t-test, *P*<0.001). The authors suggest that a deficiency in centrally available dopamine may be a significant cause of IBD fatigue, which could explain the improvement in fatigue symptoms observed with modafinil. The authors suggest that centrally available dopamine deficiency may be a significant cause of IBD fatigue, which would explain the improvement in fatigue symptoms with modafinil. Another possible explanation is that modafinil is a central nervous system stimulant approved for the treatment of sleep disorders^39^. Graff et al. (2011)^40^ 51% of whom were people diagnosed with CD. Of the participants with active Crohn’s disease (CD), 77% reported poor sleep quality. Fatigue and lack of sleep are not only highly prevalent in active disease, but they also remain significant concerns for many individuals with inactive disease Fatigue and lack of sleep are not only highly prevalent in active disease, but both remain significant concerns for many with inactive disease. Improving sleep conditions may help reduce fatigue.

### Upadacitinib and vedolizumab

Upadacitinib is an oral, reversible, small molecule JAK inhibitor designed to have increased selectivity for JAK1 compared to JAK2, JAK3, and tyrosine kinase 2, aiming to improve efficacy and safety for an improved benefit-risk profile compared to other less selective JAK inhibitors^41^. Vedolizumab is a gut-selective monoclonal antibody directed against the α4β7 integrin and is approved for the treatment of moderately to severely active ulcerative colitis and Crohn’s disease^42^.

Danese et al. (2023)^18^ performed a post hoc analysis of a large phase 3 program evaluating the effects of upadacitinib on fatigue, bowel urgency, and abdominal pain in patients with moderately to severely active ulcerative colitis. Ghosh et al. (2024)^15^ evaluated the effects of induction and maintenance therapy with upadacitinib on fatigue, quality of life, and work productivity in the phase 3 trials U-EXCEL, U-EXCEED, and U-ENDURE in patients with CRohn’s disease. Steenholdt et al. (2023)^16^ evaluated quality of life and fatigue concerning changes in disease activity during vedolizumab therapy. All three studies began with patients with active IBD and observed disease remission. All three studies observed a statistically significant impact on clinically significant symptoms of fatigue.

A possible explanation for the reduction in fatigue may be the achievement of IBD remission. Aluzaite et al. (2019)^43^ performed a detailed multidimensional assessment of fatigue in patients with IBD. They found a high prevalence of moderate to severe fatigue in patients with active disease (82.9-80.0% active).

### Limitations

This integrative review had the following limitations: a low number of articles were included, different scales were applied to assess fatigue and disease activity. No seric levels of nutrients were performed before suplementation. Furthermore, the treatment duration and follow up time after treatment were too small.

### Suggestions for future studies

A total of six scales were used to assess fatigue in the nine reviewed articles. The diversity of instruments used is a factor that makes it difficult to compare studies, showing the need for standardization of the scale, mainly because fatigue has subjective factors. Several scales to measure fatigue can make it difficult to compare results, so we suggest that future studies assessing fatigue in patients with IBD use the IBD-F self-assessment scale, as it was developed specifically to assess fatigue in patients with IBD, considering the needs and experiences of this population^9^ ^,^ ^44^. The systematic review carried out by Fan VSK and colleagues (2022) on patient-reported outcomes and fatigue in patients with IBD, recommended the use of the DII-F to assess fatigue in these patients^45^. The questionnaire is divided into three subscales: IBD-F1, consisting of 5 items to assess the severity and frequency of fatigue; IBD-F2, consisting of 30 items to assess the impact of fatigue on daily life; and IBD-F3, consisting of 5 open-ended questions to explore additional conditions related to fatigue. All subscales are scored on a scale of 0 to 4, with IBD-F3 not included in the total score. IBD-F1 options range from “no fatigue (0)” to “severe fatigue (4)”, with a total score of 0~20 points, and the higher the score, the more severe the patient’s fatigue; IBD-F2 options range from “none of the time (0)” to “all of the time (4)”, with a total score of 0~120 points, with higher scores indicating greater impact of fatigue on daily life^9^ ^,^ ^25^.

Studies evaluating pharmacological treatment for fatigue in patients with IBD are very limited, with insufficient data to identify specific drug interventions for this condition, although fatigue in IBD negatively affects quality of life and is the third most prevalent symptom^43^, the present review identified a small number of studies focused on pharmacological therapies for fatigue in this patient population. Vitamin B12 supplementation did not show promising results for reducing fatigue in patients with IBD in a clinical trial. The authors of the study do not recommend supplementation in clinical practice for the treatment of fatigue^16^. One possible reason why vitamin B12 supplementation did not reduce fatigue levels in the study by Scholten et al. (2017)^21^ is that the patients had normal plasma levels of vitamin B12, which did not indicate vitamin B12 deficiency anemia. The study by Villoria et al. (2017)^46^ analyzed the association between fatigue and vitamin B12 in patients with IBD. The mean values of micronutrients were within the normal range, and no association was found between fatigue and vitamin B12 in patients with IBD. There is a possibility that no reduction in fatigue symptoms was found due to methodological issues. Further studies to evaluate the association between vitamin B12 and fatigue are needed, including patients with vitamin B12 deficiency as the study population.

The study by Moradi et al. (2021)^17^ was the first to evaluate the use of spirulina supplementation to reduce fatigue in patients with IBD. No reduction in fatigue was observed. The authors suggest that the duration of the clinical trial and the dose applied (1g/day) may explain these findings, indicating that methodological issues may have influenced the results. They recommend further trials with higher doses and longer intervention periods.

The articles by Danese et al. (2023)^18^ and Ghosh et al. (2024)^19^ showed promising results in the reduction of fatigue with the use of Upadacitinib. This drug is a second-generation selective Janus kinase (JAK) inhibitor that targets the JAK1 enzyme and is used to achieve remission in the treatment of IBD^46^,^47^ New randomized trials involving this drug and the same target population are needed. Subsequently, it is important to conduct a systematic review and meta-analysis of the research. The study by Truyens et al. (2022)^24^ found no reduction in fatigue in IBD patients who used 5-hydroxytryptophan. They state that the lack of understanding of the multifactorial etiology of fatigue hinders the development of appropriate treatment strategies. Fatigue in IBD may not be related to low serotonin. One limitation that may have influenced the study is that the ideal dose of 5-HTP has not yet been defined, requiring further investigation. Methodological issues may have also influenced the results.

Moulton et al. (2024)^21^ studied the use of Modafinil for the treatment of fatigue in IBD. They concluded that Modafinil significantly improved IBD-related fatigue. However, further studies should be conducted, as the sample size in the study by Moulton et al. (2024)^21^ included only 10 patients. In addition, it is suggested that randomized placebo-controlled clinical trials be carried out and that they be investigated with patients with mild, moderate and severe fatigue.

It is also worth noting the reduction of fatigue in IBD with high doses of thiamine, despite the study’s limitation of including only 40 participants and a 12-week follow-up (Bager et al. 2020)^12^. The effect on fatigue was not observed with the 300 mg/day dose of thiamine^22^. The fact that the reduction of fatigue was not observed in the study with a dose of 300 mg/day of thiamine can be explained by the fact that fatigue in IBD can be the manifestation of a thiamine deficiency, probably due to a dysfunction of the active transport of thiamine within the cells, or due to structural enzymatic abnormalities. The administration of large amounts of thiamine increases blood concentration to levels where passive transport restores normal glucose metabolism in all cells, leading to a complete regression of fatigue^38^ Future multicenter, randomized controlled trials are needed to evaluate the effect of high-dose thiamine on fatigue reduction. Studies conducted in different populations diagnosed with IBD, in addition to a larger number of participants and a one-year follow-up (with monthly fatigue measurements). A systematic review with meta-analysis will also be important to evaluate the treatment of high doses of thiamine for fatigue in IBD.

Based on the study by Bager et al. (2023)^23^, where it was found that the concentration of flavin mononucleotide and riboflavin were associated with chronic fatigue in patients with IBD in remission, new studies focused on this investigation are needed so that systematic reviews with meta-analysis can be carried out in this area.

Financial investments and human resources are needed to identify pharmacological therapies for the treatment of fatigue in patients with IBD. Defining effective pharmacological therapies for fatigue in these patients may result in better physical and emotional health, better use of social relationships and employability, and reduced financial expenses for treating the problems caused by fatigue.

## Data Availability

Data usage not reported, research data not used
